# Covering reinforced staples with polyethylene glycolic acid felt-covered fibrin sealant to prevent pancreatic fistula after distal pancreatomy: a retrospective comparative study

**DOI:** 10.1186/s12893-022-01799-2

**Published:** 2022-09-22

**Authors:** Keishi Kawasaki, Tatsuya Hayashi, Makoto Takahashi, Yasuhiro Morita

**Affiliations:** Department of General Surgery, Tokyo Metropolitan Tama Medical Center, 2-8-29 Musashidai, Fuchu, Tokyo 183-8524 Japan

**Keywords:** Postoperative pancreatic fistula, Distal pancreatectomy, Polyethylene glycolic acid felt, Fibrin glue

## Abstract

**Background:**

In accordance with previous reports on the utility of polyethylene glycolic acid (PGA) felt and fibrin glue for postoperative pancreatic fistula (POPF), we usually perform distal pancreatectomy (DP) with a PGA felt reinforcement stapler when dissecting the pancreas and cover the stump with PGA felt and fibrin glue (the PPF method). In this study, we retrospectively analyzed our DP cases to compare the risk factors for POPF and the postoperative course of patients receiving the PPF method of treatment versus that of those receiving conventional treatment.

**Methods:**

A total of 127 DP procedures performed in our department between January 2008 and June 2021 were retrospectively analysed.

**Results:**

In the PPF method, grade B/C POPF rate tended to decrease, and POPF rate showed a significant decrease. The duration of drainage and the length of postoperative hospitalisation were also significantly shorter with the PPF method. The risk of grade B/C POPF significantly decreased with the PPF method if the pancreas was thick (> 13.5 mm) or the patients were obese.

**Conclusions:**

The PPF method is useful for POPF in DP and is particularly effective when a thick pancreas or obese patient is involved. Removing the drainage tube early in the PPF method may lead to early discharge.

## Introduction

Postoperative pancreatic fistulas (POPF) are the most frequent complication of distal pancreatectomy (DP) and occur in 13–64% of cases [1–3]. POPF often cause various problems leading to wound infection, intra-abdominal abscess, haemorrhage, and sepsis, resulting in a longer hospital stay and higher medical costs. Thus, the best approach to preventing POPF should be considered when performing a pancreatectomy. Many surgeons attempt to control POPF by performing various surgical techniques, but the ensuing results have mostly been unsuccessful in alleviating the problem [4, 5].

Polyethylene glycolic acid (PGA) felt is a soft, flexible, absorbable, and nonwoven material that is widely used for tissue reinforcement [6–8]. Its adhesion to tissue is the greatest when used with a fibrin sealant. Herein, we examined a combination therapy consisting of reinforcement mesh, PGA felt wrapping, and fibrin sealant application (PPF method) to evaluate the efficacy of this method in preventing POPF after DP. In this study, we retrospectively analysed our DP cases to compare the risk factors for POPF and the postoperative course of patients receiving the PPF method of treatment versus that of those receiving conventional treatment.

## Methods

### Patient demographics and clinical presentations

Between January 2008 and June 2021, 127 consecutive patients underwent DP under general anaesthesia at the Tokyo Metropolitan Tama Medical Center. Patient data were retrieved from prospectively maintained databases and included baseline patient characteristics such as demographic data, preoperative risk factors, comorbidities, operative characteristics, and postoperative outcomes.

All patients routinely underwent a preoperative enhanced computed tomography (CT). Imaging findings were assessed for main pancreatic duct (MPD) size, pancreatic thickness, and pancreatitis. Pancreatic duct dilatation was defined as MPD diameter > 3 mm on CT, a thick pancreas was defined as thickness > 13.5 mm, and pancreatitis was defined as peripancreatic infiltration or fluid collection on CT. The cutoff level of continuous variables was set based on Youden’s index using receiver operating characteristic (ROC) analysis of age, body mass index (BMI), MPD diameter, pancreatic thickness, operation time, and intraoperative blood loss. The texture of the pancreas was classified as soft or hard according to the operating surgeon’s discretion. POPF was evaluated using the International Study Group for Pancreatic Fistula (ISGPF) definition [9]. We defined delayed gastric emptying (DGE) as the inability to return to a standard diet by the end of the first postoperative week and included prolonged nasogastric intubation [10]. Chyle leak was defined as a triglyceride level in the drain higher than 110 mg/dl on or after postoperative day 3 [11].

All surgeries were performed after obtaining informed consent from the patients. This study was performed in accordance with the guidelines of the Declaration of Helsinki and its later amendments.

### Surgical techniques

For retrospective analysis, the stump closure method was selected at the discretion of the surgeon; however, the method of closing the pancreatic stump has generally changed over time.

**Fig. 1:**
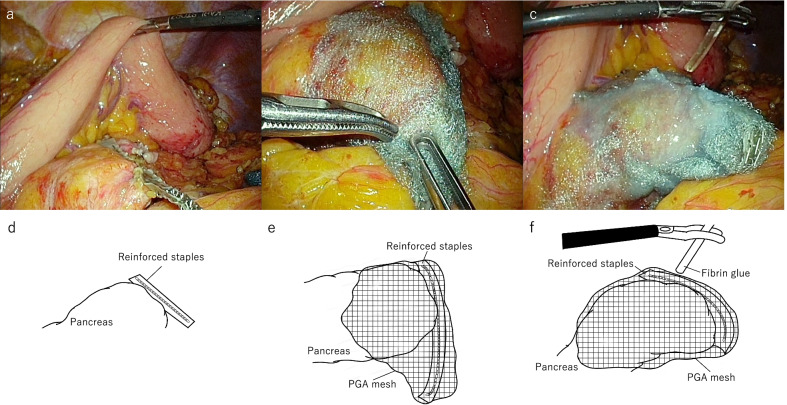
Patients in the PPF group underwent pancreatectomy with a PGA mesh reinforced stapler for pancreatic transection (**a**: photo, **d**: schema). Additionally, the PGA mesh was tightly wrapped around the pancreatic stump (**b**: photo, **e**: schema) where fibrin glue was applied from thin tube (**c**: photo, **f**: schema)

Patients in the PPF group underwent pancreatectomy with a PGA mesh reinforced stapler for pancreatic transection. Additionally, the PGA mesh was tightly wrapped around the pancreatic stump where fibrin glue was applied (Fig. 1). The type of cartridge was selected based on the thickness and texture of the pancreas by each surgeon during the operation.

### Statistical analysis

Categorical variables were expressed as numbers (%) and were compared using the chi-square test or Fisher’s exact test, as appropriate. Continuous variables were expressed as medians (interquartile range [IQR]) and were compared using Wilcoxon’s rank sum test. Statistical significance was set at P < 0.05. All statistical analyses were performed using the commercially available software JMP^®^ 13 (SAS Institute Inc., Cary, NC, USA).

## Results

### Patient demographics and clinical presentations

Between January 2008 and June 2021, 127 consecutive patients underwent DP. The median age of all patients was 70 years (IQR: 61–77 years), and the median BMI was 22.2 kg/m^2^. Examination showed that 46 patients (36.2%) had a soft pancreas, and 92 (72.4%) had a diagnosis of malignancy. The remaining 35 patients (27.6%) were diagnosed with benign disease, including 12 (9.4%) cases of intraductal papillary mucinous neoplasms, ten (7.8%) cases of neuroendocrine tumour, three (2.4%) cases of mucinous cystic neoplasm, three (2.4%) cases of serous cystic neoplasm, and seven (5.5%) cases of other diseases. The surgical procedures included open DP (ODP) in 65 patients (51.2%), laparoscopic DP (LDP) in 61 patients (48.0%), and robot-assisted DP (RDP) in one patient (0.8%). The pancreatic stump was treated using the PPF method in 17 patients (13.4%), reinforced staplers alone in 29 patients (22.8%), staplers alone in 68 patients (53.5%), and MPD ligation in 13 patients (10.2%). The median operation time in all cases was 334 min (IQR: 275–394 min), and the median intraoperative blood loss was 340 ml (IQR: 150–870 ml). Prefiring compression was done in 85 patients, including 17 using the PPF method. Grade B/C POPF occurred in 56 patients (44.1%) (Table 1).

**Table 1:** Patient demographics and clinical presentations

Variables	Total (n = 127)
Age, years (median (range))	70.0 (61.0–77.0)
BMI, kg/m^2^ (median (range))	22.2 (20.4–24.9)
Histological grade (benign vs. malignancy)	92/35
Pancreatic texture (soft vs. hard)	46/81
Minimal invasive surgery (open vs. lap/robot)	65/62
MPD size, mm (median (range))	2.9 (2.3–3.5)
Thickness of resection, mm (median (range))	13.5 (10.9–15.9)
Pancreatitis (yes vs. no)	63/64
Operation time, min. (median (range))	334.0 (275.0–394.0)
Blood loss, ml (median (range))	340.0 (150.0–870.0)
Pre-compression (yes vs. no)	85/42
PPF method (yes vs. no)	17/110
POPF (no-POPF, BL vs. grade B/C POPF)	71/56

*BMI* body mass index, *MPD* main pancreatic duct, *POPF* postoperative pancreatic fistula, *BL* biochemical leak

### Comparison of the factors related to grade B/C POPF

Grade B/C POPF developed in 56 (44.1%) DP’s, and the all the cases were Grade B. There were no cases of POPF-related death. Based on Youden's index using ROC analysis of grade B/C POPF, the 127 patients were divided into two groups based on age, BMI, MPD diameter, pancreatic thickness, operation time, and intraoperative blood loss. Most of the patients who developed a fistula after DP were obese. High BMI was associated with a higher fistula rate. (63.4% vs. 83.9%; P = 0.010).

Pancreatic characteristics, including duct size and gland texture, may be used to identify patients at high risk of fistula development following DP. Large MPD (≥ 3 mm) and thick pancreas (≥ 13.5 mm) were also associated with a higher fistula rate (38.0% vs. 57.1%; P = 0.032 and 42.3% vs. 60.7%; P = 0.039, respectively). There was no significant difference in the pancreatic texture in the present cohort.

The intraoperative factors were also examined. An intraoperative blood loss > 800 ml was associated with a higher fistula rate (12.7% vs. 46.4%; P < 0.001). Minimally invasive procedures, including laparoscopic and robotic surgery and prefiring compression, were not significantly associated with grade B/C POPF formation (P = 0.096 and P = 0.452, respectively) (Table 2).

**Table 2:** Comparison of the factors related to grade B/C POPF

Variables	No-POPF (n = 71)	Grade B/C POPF (n = 56)	*P* value
Age, years (≥ 75 vs. < 75)	24/47	14/41	0.394
BMI, kg/m^2^ (≥ 20.5 vs. < 20.5)	45/26	47/9	0.010
Histological grade (benign vs. malignant)	20/51	15/41	0.863
Pancreatic texture (soft vs. hard)	26/45	20/36	0.916
Minimal invasive surgery (open vs. lap/robot)	30/41	32/24	0.096
MPD size, mm (≥ 3 vs. < 3)	27/44	32/24	0.032
Thickness of resection, mm (≥ 13.5 vs. < 13.5)	30/41	34/22	0.039
Pancreatitis (Yes vs. No)	33/38	26/30	0.427
Operation time, min. (≥ 445 vs. < 445)	5/66	9/47	0.107
Blood loss, ml (≥ 800 vs. < 800)	9/62	26/30	< 0.001
Pre-compression (yes vs. no)	49/21	35/20	0.452
PPF method (yes vs. no)	13/58	4/52	0.067

*POPF* postoperative pancreatic fistula, *BMI* body mass index, *MPD* main pancreatic duct

### Risk factors associated with POPF

Univariate analysis revealed that a soft pancreas (odds ratio [OR]: 6.03; P = 0.020), large blood loss (OR: 7.02; P = 0.003), and non-application of the PPF method (OR: 0.18; P = 0.023) were independent predictors of grade B/C POPF. Multivariate analysis revealed that only excess blood loss (OR: 7.64; P < 0.001) was an independent predictor of grade B/C POPF (Table 3). These findings suggested that the non-application of the PPF method could be a risk factor for POPF.

**Table 3:** Risk factors associated with grade B/C POPF

	Univariate analysis		Multivariate analysis	
	Hazard ratio	P-value	Hazard ratio	P-value
Age, years (≥ 75 vs. < 75)	1.220 (0.489–3.041)	0.669		
BMI, kg/m^2^ (≥ 20.5 vs. < 20.5)	2.612 (0.958–7.123)	0.061		
Histological grade (benign vs. malignant)	3.664 (0.858–15.648)	0.080		
Pancreatic texture (soft vs. hard)	6.027 (1.336–27.192	0.020	2.356 (0.967–5.740)	0.059
Minimal invasive surgery (open vs. lap/robot)	1.262 (0.403–3.946)	0.690		
MPD size, mm (≥ 3 vs. < 3)	1.556 (0.640–3.784)	0.329		
Thickness of resection, mm (≥ 13.5 vs. < 13.5)	2.135 (0.842–5.418)	0.110		
Pancreatitis (Yes vs. No)	1.152 (0.440–3.017)	0.774		
Operation time, min. (≥ 445 vs. < 445)	2.155 (0.481–9.648)	0.315		
Blood loss, ml (≥ 800 vs. < 800)	7.019 (1.984–24.836)	0.003	7.643 (2.889–20.217)	< 0.001
Pre-compression (yes vs. no)	1.641 (0.592–4.548)	0.341		
PPF method (yes vs. no)	0.177 (0.040–0.788)	0.023	0.382 (0.108–1.352)	0.136

*POPF* postoperative pancreatic fistula, *BMI* body mass index, *MPD* main pancreatic duct

### Comparison of postoperative outcomes in the PPF and conventional groups

The PPF and conventional groups consisted of 17 patients (13.4%) and 110 patients (86.6%), respectively. The grade B/C POPF rate (13.9% vs. 24.0%; P = 0.006) tended to be lower in the PPF group. The rates of other surgical complications, including DGE, post-pancreatectomy haemorrhage, and chyle leakage, were similar between the groups. In addition, the rates of postoperative intra-abdominal fluid collection were significantly lower in the PPF group. Moreover, compared with the control group, the PPF group had a significantly shorter median length of drainage tube insertion and hospital stay (22 days vs. 8 days; P < 0.001 and 28 days vs. 12 days; P < 0.001, respectively) (Table 4).

**Table 4:** Comparison of postoperative outcomes in the PPF and conventional group

Variables	Former (n = 110)	PPF (n = 17)	*P* value
POPF (no-POPF vs. BL, grade B/C POPF)	35/75	10/7	0.030
Grade B/C POPF (no-POPF, BL vs. grade B/C POPF)	58/52	13/4	0.067
Intra-abdominal fluid collection (yes vs. no)	2/108	5/12	< 0.001
Delayed gastric emptying (yes vs. no)	13/97	0/17	0.135
Chyle leak (yes vs. no)	19/91	1/16	0.230
PPH (yes vs. no)	2/108	0/17	0.575
Duration of drainage tube insertion, days	22.0 (10.0–38.0)	8.0 (6.0–14.5)	< 0.001
Length of hospital stay, days	28.0 (14.8–40.3)	12.0 (9.5–19.0)	< 0.001

*POPF* postoperative pancreatic fistula, *BL* biochemical leak, *PPH* post-pancreatectomy haemorrhage

### Comparison of postoperative outcomes in POPF high-risk cases in the PPF and conventional groups

**Fig. 2:**
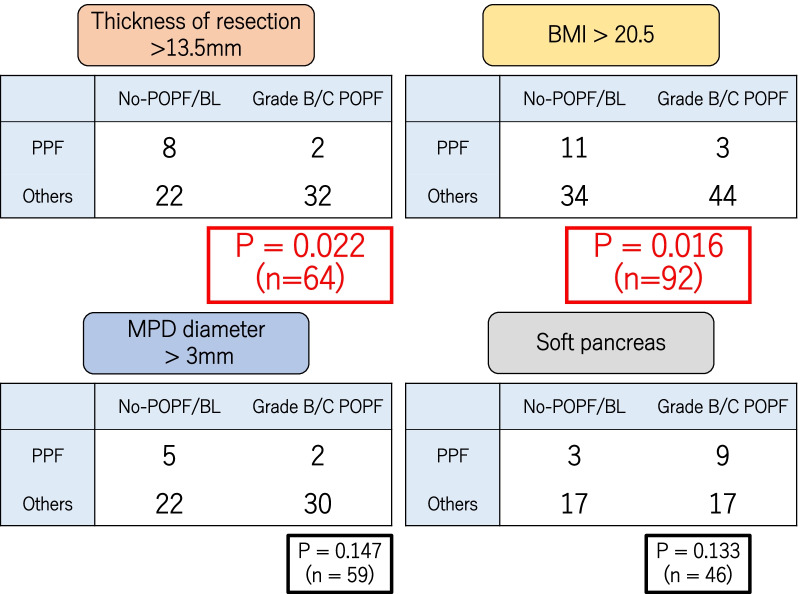
Grade B/C POPF occurrence did decrease significantly among patients in the PPF group with a thick pancreas (> 13.5 mm) or high BMI (P = 0.022 and P = 0.016, respectively)

Grade B/C POPF occurrence did not decrease uniformly in all cases treated with the PPF method. However, it decreased significantly among patients in the PPF group with a thick pancreas (> 13.5 mm) or a high BMI (P = 0.022 and P = 0.016, respectively). Grade B/C POPF was also evaluated in patients with a large MPD diameter and a soft pancreatic texture, but no significant difference was found between these groups (Fig. 2).

### Comparison of postoperative outcomes between the PPF and reinforced stapler-alone groups

**Fig. 3:**
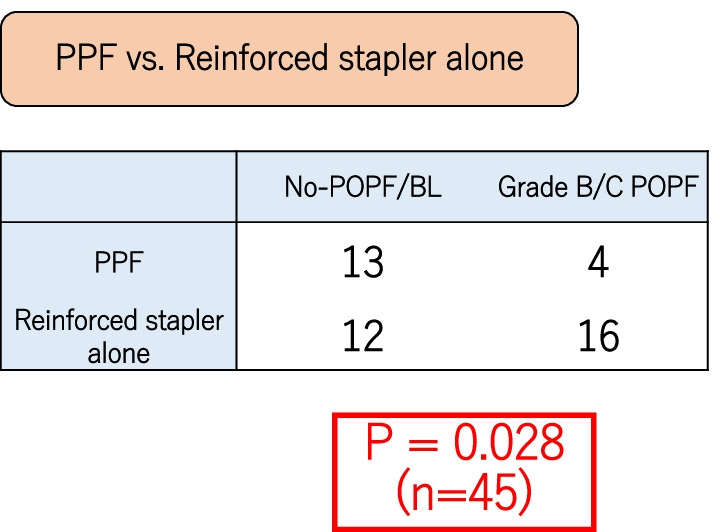
Grade B/C POPF occurrence decreased to a significantly greater extent in the PPF group than in the reinforced stapler-alone group (P = 0.028)

A previous study revealed that the use of a stapler with PGA mesh reinforcement independently decreased the risk of post-DP pancreatic fistula formation [12, 13]. It is possible that only PGA reinforcement without additional PGA mesh wrapping or fibrin glue was sufficient to prevent post-DP POPF. Thus, we compared the reinforced stapler-alone and PPF methods and found that grade B/C POPF occurrence decreased to a significantly greater extent in the PPF group than in the reinforced stapler-alone group (P = 0.028). (Fig. 3).

## Discussion

Previous studies have reported several methods for preventing POPF, including MPD ligation [14], staple closure [15, 16], pancreatico-intestinal anastomosis [17], and somatostatin analogues [18, 19]. However, the effectiveness of all these methods remains unclear.

Fibrin sealant is widely used to prevent POPF in patients with DP [20, 21]. Suzuki et al. showed that intraoperative fibrin sealant was effective in preventing POPF after DP. They revealed that POPF decreased significantly in the fibrin sealant group compared to that in the control group (15% vs. 40%). Ohwada et al. reported the fibrin sealant sandwich technique. They inserted fibrin sealant between the dorsal and ventral edges of the pancreatic stump. They reported that POPF decreased significantly in the sandwich technique group compared to that in the simple sealing group (9% vs. 27%).

PGA felt (Neoveil; Gunze), a bioabsorbable recombinant membrane made of synthetic polymer with a cellulose-like structure, is frequently used with fibrin sealant in thoracic surgery. Several studies have revealed that the application of layers of PGA felt on the remnant pancreatic stump and the use of fibrin glue with layers of PGA mesh are effective in preventing post-DP pancreatic fistula development [22, 23].

Previous studies have reported that the use of a reinforced stapler [12, 13] in pancreatic transection decreases the risk of POPF formation. Kawaida et al. reported that POPF rates with and without a reinforcement stapler were 3.6% and 13.5%, respectively [12]. The present study also examined whether a reinforced stapler alone was sufficient but found that the grade B/C POPF incidence was significantly lower in the PPF method group than in the reinforced stapler-alone group. In addition, in recent years, two RCTs have reported that the reinforcement stapler alone is not sufficient for POPF prevention in DP, which also suggests that further technical ingenuity is required for pancreatic stump closure with the reinforcement stapler alone [24, 25].

The present study focused on patients with a high risk of POPF characterised by a soft pancreas, wide MPD, obesity, or thick pancreatic resection. We assumed that pancreatic fistulas were less likely to develop if a method for treating the pancreatic stump was used with pancreatic resection in patients with a low risk of pancreatic fistula formation, such as those with a hard or thin pancreas, a normal-sized MPD, or normal BMI. Therefore, our investigation of pancreatic resection in patients with a high risk of POPF found that the PPF method is useful in patients with a thick pancreas or obesity, suggesting that using the PPF method in patients with a high risk of POPF may lead to better outcomes.

**Fig. 4:**
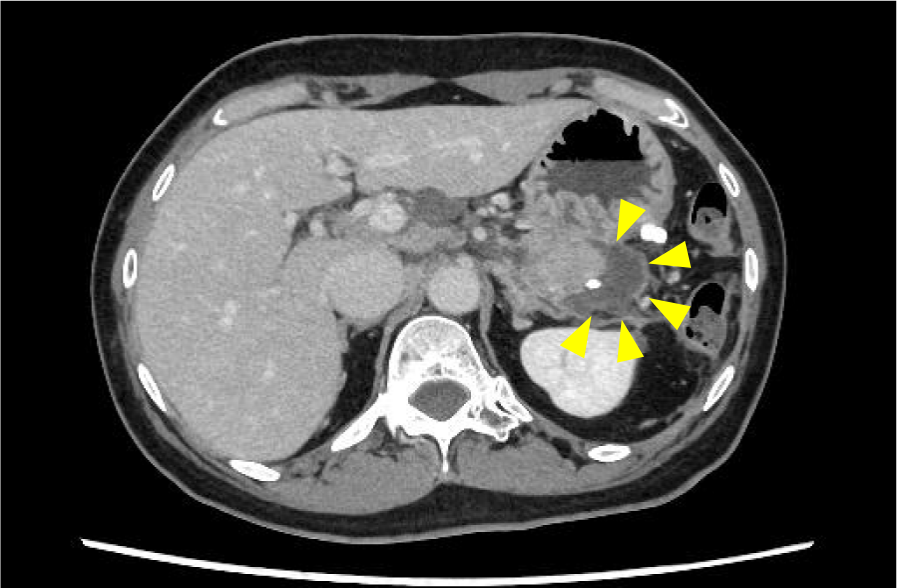
The PPF method demonstrated significantly delayed postoperative fluid collection in the pancreatic stump (arrowhead). However, all the cases improved with conservative treatment, such as antibiotic treatment, and did not require surgical intervention

In addition, the PPF method significantly demonstrated delayed postoperative fluid collection in the pancreatic stump. However, all the patients improved with conservative treatment, such as antibiotic treatment, and did not require surgical intervention (Fig. 4). These results suggest that even if fluid collection in the pancreatic stump, a form of pancreatic fistula, is observed with the PPF method, conservative treatment can sufficiently improve the patient’s condition to enable early removal of the drainage tube. The PPF method is particularly effective in dissecting a thick pancreas, and early removal of the drainage tube in the PPF method has the potential to promote early discharge.

A previous study reported a new method of using PGA felt in conjunction with fibrin sealant for pancreaticojejunal (PJ) anastomosis in pancreaticoduodenectomies, however, its utility remains controversial [8, 26]. We hypothesised that the covering of the PJ anastomotic line itself is probably a reason for the disappointing outcomes, i.e., completely wrapping and sealing the PJ anastomosis can cause exudates, including pancreatic juice from the small ductal branches of the cut surface of the pancreatic remnant, to collect leading to PJ anastomosis infection and eventually POPF formation.

In conclusion, the present study found that the PPF method may be useful for preventing post-DP pancreatic fistula and is particularly effective when dissecting a thick pancreas or performing DP in an obese patient. Moreover, early drainage tube removal in the PPF method may lead to early discharge.

## Data Availability

The datasets used and/or analyzed during the current study available from the corresponding author on reasonable request.

## Abbreviations

POPF: Postoperative pancreatic fistula
DP: Distal pancreatectomy
PGA: Polyethylene glycolic acid
CT: Computed tomography
MPD: Main pancreatic duct
ROC: Receiver operating characteristic
BMI: Body mass index
ISGPF: International study group for pancreatic fistula
DGE: Delayed gastric emptying
IQR: Interquartile range
LDP: Laparoscopic distal pancreatectomy
OD: Odd ratio
RDP: Robot-assisted distal pancreatectomy
PJ: Pancreaticojejunal
